# Cutaneous metastases in renal cell carcinoma: a case report

**DOI:** 10.4076/1757-1626-2-7948

**Published:** 2009-08-25

**Authors:** Miguel Angel Arrabal-Polo, Salvador A Arias-Santiago, Jose Aneiros-Fernandez, Pilar Burkhardt-Perez, Miguel Arrabal-Martin, Ramon Naranjo-Sintes

**Affiliations:** Dermatology and Urology Services, San Cecilio University HospitalAvenida Doctor Oloriz. PC: 18012. GranadaSpain

## Abstract

Renal cell carcinoma is the most common form of malignant renal tumour and is extremely lethal. About 25% of the patients develop metastasis at the time of diagnosis, and in many cases during the course of the disease, affecting the lung, lymphatic ganglions, liver, and bone, with skin metastases being quite rare.

A 73-year-old patient, who had undergone surgery for adenocarcinoma in the left kidney 10 years previously, visited the dermatological service due to the appearance of recent, rapidly-developing lesion at the back of his neck. It was decided to remove it surgically. The histological study confirmed clear cell carcinoma that was probably of renal origin. A computed tomography scan was performed on the thorax and abdomen, and lesions were observed that were compatible with metastasis in the right kidney and left lung. Treatment with a multikinase angiogenesis inhibitor (sunitib) was started.

Due to the late development of the skin metastases and those in other regions that worsen the prognosis, these patients must be subjected to long-term clinical observation. Urologist should pay attention to cutaneous lesion appearing in these patients as in many times they look like benign lesion.

## Introduction

Renal Cell Carcinoma (RCC) currently accounts for 90% of all renal tumours, and is the most lethal of urological tumours. Its frequency has increased by 2.5% per annum. Thanks to the increase in image diagnosis (ultrasound, CT scan, MRI) in recent years, these tumours are easier to diagnose at an early stage of the disease. However, it continues to be a highly lethal tumour in the event of the disease being widely disseminated at the time of diagnosis.

Approximately 25% of patients diagnosed for RCC are shown to have metastasis at the time of the diagnosis, and these are more frequent in the following order: lungs, lymphatic ganglions, bone, liver, contralateral kidney, adrenal and ipsilateral glands, brain and other less frequent locations such as the skin [1,2].

A case study is presented consisting of a male patient aged 73 years who had undergone a left radical nephrectomy 10 years ago for grade 3 Fuhrman’s renal adenocarcinoma. Skin lesion had developed 2 months ago on the patient’s neck, and a histological diagnosis confirmed the presence of clear cells, showing positive for EMA, CEA and CD10 [3].

## Case presentation

**Figure 1.**
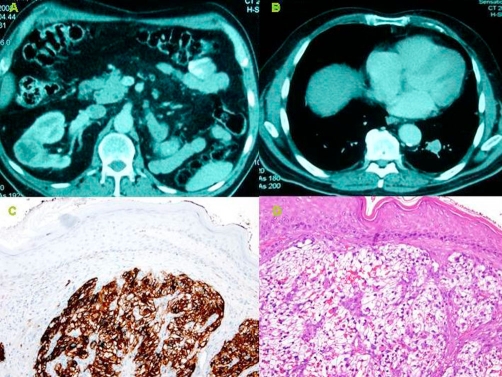
**(A)** A 3.7 cm heterogeneous image in the middle of the right kidney. **(B)** A 2.5 cm nodule in the lower lobule in left lung. **(C)** The IHC test showed positive for CD10. **(D)** Goups of irregular cells with cytological irregularities (large nuclei and prominent nucleoles) and clear cytoplasm are observed in dermis (Hematoxylin-Eosin).

Caucasian and Spanish patient aged 73 years with minor renal failure on whom a left radical nefrectomy for renal adenocarcinoma had been performed 10 years ago in the Urology Unit. The patient was referred to the Dermatological Unit for an asymptomatic lesion at the back of his neck that had developed 2 months previously. The examination revealed the presence of a tumour with a firm consistency, bluish-red in colour, with a somewhat hyperkeratous surface and maximum diameter of 2.5 cm, which was initially diagnosed as a possible angioma. Surgical treatment was indicated due to the late development of the lesion and its rapid growth. The histopathological study revealed the presence of groups of irregular cells with cytological irregularities (large nuclei and prominent nucleoles) and clear cytoplasm (Figure 1D). The IHC (immunohistochemistry) (Figure 1C) test showed positive for CD10, EMA, CEA, vimentin, and pancytokeratin and it was therefore diagnosed as a Clear Cell Carcinoma that was probably of renal origin, taking into account the patient’s history.

In view of the histological results, it was decided to request a CT scan of the abdomen and pelvis which showed an heterogeneous image with a maximum diameter of 3.7 cm in the middle of the right kidney (Figure 1A), suggesting metastasis of a renal adenocarcinoma in the kidney that had been removed and a nodule with a maximum diameter of 2.5 cm in the lower lobule of the left lung that seemed to correspond with another metastasis of the same adenocarcinoma (Figure 1B). Those findings were not present in the CT scan of the abdomen and pelvis performed on the patient 12 months previously.

Due to the presence of multiple metastases, it was decided to refer the patient to the Cancer Unit for evaluation and the administration of medication with angiogenesis/ multikinase inhibitors.

## Discussion

Owing to the clinical and evolutive particularities of the RCC, in certain cases diagnosis of this condition is made at a late stage of the disease when the cancer is widespread and has metastasised in different areas of the body. The development of skin metastases accounting for between 1 and 3% of all metastases. The most usual location of skin metastases in these patients is the scalp and face, and they are usually single lesions that grow rapidly, are bluish-red in colour and sometimes pulsating [1,4], differential diagnosis performed macroscopically is necessary to rule out angioma, cutaneous horns, basal cell carcinoma and microscopically to rule out xanthoma, xanthelasma, sebaceous adenoma, sebaceous carcinoma, sebaceous epithelioma, balloon cell nevi, clear cell hydro adenomas and other skin pathologies characterised by the presence of clear cells [3,5]. The performing of IHC techniques currently makes it possible to conduct differential diagnosis of those lesions. EMA, CEA, CD-10 and recently, RCC-MA (positive in 60% of all RCC skin lesions) are all markers that suggest skin metastases of renal origin [3,5].

In most of the cases published regarding patients with RCC, the development of skin metastases takes place within six months to five years of the initial diagnosis and after performing the nefrectomy [1,2,6], except in some cases in which RCC has been diagnosed after removing the skin lesion [3,4]. In our case, the development of skin metastases occurred ten years after the nefrectomy, which is not common during the natural course of the disease. We should highlight the importance of a precise histological diagnosis to permit the correct identification of the skin lesion in order to complete an extension study, since in up to 75% of cases, concomitant organic metastases develop. In this way, it was possible to diagnose other metastatic lesions in the lung and contralateral kidney that had not been detected during routine patient monitoring [7,3].

Treatment of metastatic renal adenocarcinoma consists of a combination of surgical treatment (radical nephrectomy) and angiogenesis/multikinase inhibitors (sunitinib or sorafenib). However, treatment of single skin lesions is usually surgical [3], except in certain cases in which radiotherapy is an option [8,9]. In this case, due to the late development of multiple metastases following the nefrectomy, it was decided to operate on the patient and remove the skin lesions followed by treatment with multikinase inhibitors that have shown a significant increase in the possibilities of survival compared to other medical therapies (Interferon or Interleukins) in treating renal cell carcinoma [4].

In short, we wish to emphasise the peculiar natural history of renal adenocarcinoma, in addition to the need for exhaustive monitoring of these patients even up to ten years after the initial diagnosis, due to the development of late metastases that worsen the clinical prognosis and reduce life expectancy, showing an average survival rate after diagnosing metastasis, of between 7 and 32 months [2,10].

## Abbreviations

CT: computerized tomography
IHC: immunohistochemistry
MRI: magnetic resonance image
RCC: renal cell carcinoma
